# End of life in the intensive care unit: should French law be adapted?

**DOI:** 10.1186/2110-5820-4-6

**Published:** 2014-02-25

**Authors:** René Robert, Laurence Salomon, Lise Haddad, Jean-Pierre Graftieaux, Béatrice Eon, Didier Dreyfuss

**Affiliations:** 1 Service de Réanimationmédicale, CHU de Poitiers, University of Poitiers, Poitiers, France; 2 Unité de Recherche Clinique, Fondation Ophtalmologique Adolphe de Rothschild, 25 rueManin, 75019 Paris, France; 3 7 rue Théodore de Banville, F-75017 Paris, France; 4 Départementd’anesthésie-réanimation, CHU Reims, hôpital Robert Debré, 51100 Reims, France; 5 Service de réanimationd’urgence et médicale, CHU de Marseille, hôpital de la Timone, F-13385 Marseille, cedex 05, France; 6 Service de réanimationmédicochirurgicale, CHU Louis Mourier, Université Paris Diderot, Sorbonne Paris Cité, UMRS 722, F-92701 Colombes cedex, F-75018 Paris, France

**Keywords:** Euthanasia, Palliative care, End of life, Intensive care unit

## Abstract

**Background:**

Longstanding concerns regarding end of life in the ICU led in France to the publication of guidelines, updated in 2009, that take into account the insights provided by a recent law (Leonetti’s law) regarding patients’ rights. After the French President asked a specific expert to review end of life issues, the French Intensive Care Society (SRLF) surveyed their members (doctors and paramedics) about various aspects of end of life in the ICU.

**Methods:**

SRLF members were invited to respond to a questionnaire, sent by Email, designed to assess their knowledge of Leonetti’s law and to determine how many caregivers would agree with the authorization of lethal drug administration in selected end of life situations.

**Results:**

Questionnaires returned by 616 (23%) of 2,700 members were analyzed. Most members (82.5%) reported that they had a good knowledge of Leonetti’s law, which most (88%) said they have often applied. One third of respondents had received ‘assisted death’ requests from patients and more than 50% from patients’ relatives. One quarter of respondents had experienced the wish to give lethal drugs to end of life patients. Assuming that palliative care in the ICU is well-managed, 25.7% of the respondents would approve a law authorizing euthanasia, while 26.5% would not. Answers were influenced by the fear of a possible risk of abuse. Doctors and nurses answered differently.

**Conclusion:**

ICU caregivers appear to be well acquainted with Leonetti’s law. Nevertheless, in selected clinical situations with suitable palliative care, one quarter of respondents were in favor of a law authorizing administration of lethal drugs to patients.

## Background

The French Intensive Care Society (SRLF) issued its first recommendations concerning withholding and withdrawal of life support in 2002 [1]. They stemmed from ethical considerations regarding intensive care patients in whom certain life-sustaining treatments appeared unwarranted in terms of expected benefits, not only in survival but also in quality of life [2]. These recommendations received legal support in 2005 by the passing of a specific law (Leonetti’s Law) and by the decrees covering its application which recognize the right to withhold or withdraw futile medical care [3-5], filling the gap between two potentially contradictory statements of the code of medical ethics: one stipulating that prolongation of agony is forbidden and the other prohibiting the hastening of death [6]. The SRLF recommendations were updated in 2009 to include, among other things, items specific to the law [7]. On the occasion of media debates in 2008 regarding euthanasia, and when another law was tabled in the Senate on 8 June 2012 regarding medically assisted death and access to palliative care, the SRLF, through press releases, voiced its commitment to well-managed palliative care and underscored the need to heighten awareness and to improve application of Leonetti’s law, which offers a legal framework covering the great majority of end of life situations in intensive care [8,9]. In its 2012 position statement, the SRLF considered that the problem of euthanasia arises infrequently in intensive care, emphasized the dangers of an emotional debate on the subject and called for a nationwide study of the issue [9]. In the context of the mission on end of life questions entrusted to Professor Didier Sicard, previous president of the French Ethics Committee, by the French President François Hollande, the SRLF, through its ethics commission, wished to delve deeper into this issue and not to limit its stance to the views of high-profile participants in the debate, but to sound out the opinions of intensive care personnel - doctors and paramedics -on questions concerning end of life in the ICU.

## Methods

The members of the SRLF ethics commission drew up a questionnaire comprising five demographic items and 22 questions. The questions concerned knowledge of Leonetti’s law and its implementation and were designed to determine the proportion of caregivers who, in certain end of life situations, would be in favor of the administration of lethal drugs, and why (Additional file 1).

The purpose of the questionnaire was twofold:

•to analyze ethical considerations using figures even though these data may not strictly correspond with real-life situations (questionnaires versus real cases)

•to propose an educational approach to these ethical considerations to personnel who work in ICUs and are therefore concerned with end of life issues

Special care was taken to formulate neutral questions. The questionnaire was sent to 2,700 SRLF members electronically, and recipients were given three weeks to respond electronically, during which time they were sent three electronic reminders.

### Statistical analysis

The vast majority of the epidemiological data collected were qualitative. The results were expressed as the total number of answers and as a percentage. The minimum number of respondents was not calculated before the analysis of the answers. Comparisons were made using the Chi-squared test between two populations of respondents: doctors and paramedics. A *P*-value below 0.05 was taken as significant.

## Results

Of the 2,700 SRLF members who were sent the questionnaire, 707 (26%) replied, and the answers from 616 (23%) of these were usable and constituted the database. These respondents comprised 454 (74%) doctors, 48 of whom were interns, and 162 (26%) paramedical staff, who included 131 nurses, 24 healthcare managers, two healthcare support workers, one physiotherapist and four psychologists. The demographic data of the respondents are given in Table 1. Most respondents (82.5%) reported being fully acquainted with Leonetti’s law, although familiarity was less frequent among paramedics than among doctors (Table 2). Most paramedics (72%) reported that they personally knew the law, but surprisingly, only 39% considered that their profession in general did; a view shared by doctors who reckoned that paramedics had a poor knowledge of the law (Table 2).

**Table 1:** Demographic data

	**Total n = 616**	**Doctors n = 454**	**Paramedics n = 162**
Age			
<30	14.3	1.5	28
30 to 40	44.6	47.3	44
40 to 50	24.7	29.3	20
50 to 60	12.2	15.8	7
>60	4.2	6.9	1
Sex ratio	59/41	72/28	20/80
Years spent working in intensive care			
<1	4.7	3.4	3
1 to 5	30.8	24.6	33
5 to 10	23.7	22.9	32
10 to 20	24.4	28.3	22
>20	16.4	31.4	10
University hospital	61/28	60/30	58/27
Hospital/hospital	11	10	15

**Table 2:** Knowledge of Leonetti’s law (figures in parentheses are percentages)

	**Total n = 616**	**Doctors n = 454**	**Paramedics n = 162**	***P***
Good knowledge of Leonetti’s law	508 (82.5)	315 (87)	152 (71.7)	0.001
Very good/good	110 (17.9)/398 (64.6)	92 (23)/264 (65)	189.9/134 (61.7)	0.001
Poor/very poor	105 (17)/3 (0.5)	49 (12)/2 (0.5)	56 (27.8)/1 (0.2)	
Estimation of knowledge of Leonetti’s law by the doctors in the unit	507 (82.3)	331 (83.4)	138 (85)	NS
Estimation of knowledge of Leonetti’s law by the paramedics in the unit	215 (34.9)	138 (34)	63 (39)	0.05

The vast majority of respondents thought that Leonetti’s law was often applied in end of life situations and allowed life-sustaining treatments to be withheld or withdrawn. This feeling was reported significantly more by doctors than by paramedics (Table 3). The difficulty most often identified in application of the law, particularly by doctors, concerned use of an outside consultant. Interestingly, paramedics reported that collective decision-making was lacking in onethird of cases and that in 20% of cases they had not even been informed of the decision taken (Table 3).

**Table 3:** Application of Leonetti’s law

	**Total n = 616**	**Doctors n = 454**	**Paramedics n = 132**	**P**
Often applied	543 (88)	422 (93)	101 (77.2)	0.001
Always and fully applied	168 (27.3)	26 (28)	31 (24.1)	NS
Difficulties encountered				
Collective decision-making difficult to achieve	18	13	31.7	0.001
No team discussion	7.3	3	19.5	0.001
Outside consultant not contacted or unavailable	73	84	46.3	0.01
Covers all end of life situations in intensive care				
(always or usually)	79.5	86.5	67.9	0.001
Allows decision-making concerning withholding or withdrawing life-sustaining treatment in most cases in your unit	81	87	68.5	0.001

An insistent or repeated request from a patient for help in dying was reported by 33% of respondents, with a significantly higher frequency among paramedics (Table 4). Such requests from the patient’s loved ones were reported in over 50% of cases (Table 4). Conversely, 74% of respondents reported that they sometimes experienced a clear and persistent opposition of the patient’s loved ones to the possibility of withholding or withdrawing life-sustaining treatments proposed by the healthcare team. Such opposition was reported equally by doctors and paramedical staff.

**Table 4:** Personal experiences in 2012

	**Total**	**Doctors**	**Paramedics**	**p**
Have you been asked by a patient to help him/her die?	33	30	40.1	<0.01
More than once	15.1	14	17.3	<0.05
Have you been asked by a patient’s loved ones to help him/her die?	56.2	59.5	49.2	NS
More than once	36.4	40	22.9	<0.05
Have you ever envisioned administering lethal drugs to a patient?	25.2	23	27.8	NS

### Administration of lethal drugs - assisted dying

One-quarter of respondents reported having envisioned administering lethal drugs to a patient. Twenty-five point seven percent were in favor of a law authorizing euthanasia in intensive care, assuming suitable palliative care is administered; 26.5% were opposed. Fears were expressed regarding potential misuse or abuse of such a law.

As for the question of a possible law authorizing the administration of lethal drugs, opinions were equally distributed in favor or against such a law both in the case of conscious patients (48.5% against versus 51.5% for) and unconscious patients (47.4% against versus 52.6% for).

Lastly, 43% of respondents (and 37% of doctors) thought that it would be technically possible to draw up a law on a request for euthanasia by conscious patients, but only 23% (23% of whom were doctors) thought this possible for unconscious patients.

In the clinical situation of an intensive care patient for whom a decision to withhold or withdraw life-sustaining treatment is taken, but whose death does not occur after a certain time, 50.3% of respondents expressed a wish for the patient’s death to come quicker, but only 9.6% were in favor of the administration of lethal drugs even if authorized by the law. No respondent reported using such drugs currently and 49.7% accepted the time to the occurrence of death, whatever its duration, provided that the patient appeared to be comfortable.

## Discussion

In this survey a large majority of respondents reported that they were well acquainted with Leonetti’s law, but that it was not necessarily optimally applied. Most respondents (80%), notably doctors (85%), thought that Leonetti’s law covered all end of life situations in intensive care. Despite this, onequarter of respondents were in favor of a law authorizing euthanasia, while admitting the difficulties associated with drawing up such a law, particularly in the case of unconscious patients.

### Knowledge and application of the law

A law tabled at the French parliament on 8 June 2012, on medically assisted dying and access to palliative care, reopened the debate on the end of life problem. Twice, in 2008 and in 2012, French intensivists, through the SRLF ethics commission, underscored the dangers of an emotional debate on euthanasia and called for a national debate on the issue [8,9]. The SRLF stressed the need to be fully acquainted with Leonetti’s law, which covers alleviation of physical and mental suffering at the end of life, while respecting patients’ dignity. The SRLF also highlighted the need to give all caregivers suitable training in the ethical approach to such situations and in end of life care. However, these position statements were not based on objective data. Since the enactment of Leonetti’s law in 2005 and of the decrees covering its application in 2006, numerous training measures have been introduced in intensive care [10]. In particular, the SRLF has updated its recommendations for clinical practices applied at the end of life, incorporating data provided by Leonetti’s law [7].

The first interesting result of the present study is that caregivers in intensive care, especially doctors, report that they are well acquainted with Leonetti’s law. Note that paramedics, although seemingly familiar individually with the law, consider that their profession in general is poorly acquainted with it. This may be linked to the fact that at interdisciplinary meetings, the mechanisms leading to the decision to withhold or withdraw life-sustaining treatment are unclear, in contrast to the quite good knowledge of the decision-making individuals. It is also reassuring to think that Leonetti’s law is not only known, but is also applied in the overwhelming majority of cases. This is not surprising because intensivists have long been aware of end of life problems [2]. In France, the first recommendations for an ethical debate on end of life situations were made over ten years ago [1]. However, complete conformity with the law seems difficult to achieve. Although the questionnaire is not precise enough to analyze points of disagreement, the difficulty most often reported by doctors related to consultation with an outside expert. This is unsurprising because the law makes no distinction between the decisions to withhold or withdraw life-sustaining treatment. It is easy to imagine the organizational difficulties that arise in seeking an outside opinion for every decision to withhold treatment. The SRLF issued advice on the role of this outside consultant that is peculiar to France and is not applied in other countries [11].

### The temptation to implement euthanasia

Few countries authorize the injection of lethal drugs with a view to terminating end of life suffering. In Switzerland, patients can request assisted suicide. In the Netherlands, Belgium and Luxembourg, euthanasia is possible, when requested by the patient [12]. In all these cases, the procedures to be followed are strictly imposed by the authorities. Surprisingly, the European countries that allow euthanasia have not issued a law on end of life which could compare with French legislation. In many other countries, there is open debate on palliative care and the administration of lethal drugs. The recommendations of the American Society of Critical Care Medicine are similar to the French recommendations in that ‘allowing to die’ is possible but ‘killing’ is not [13]. Despite reporting good knowledge of Leonetti’s law and of its routine application, onequarter of respondents in our survey seemed tempted by the administration of lethal drugs in certain end of life situations, in response to a request clearly and repeatedly made by the patient. Such a request for assisted dying is also frequently made by the patient’s loved ones. There is no unequivocal way of interpreting such requests, and a genuine wish to die should be interpreted with care. Decision-making capacity and ability to give informed consent vary among intensive care patients, including those with an apparently unaltered state of consciousness [14]. Likewise, understanding of explanations given to the families of intensive care patients is not always optimal and is often impaired by the high levels of anxiety and depression attendant upon these situations [15]. In addition, respondents’ views on legalizing euthanasia are clearly complex. The question, formulated in different ways, is posed several times in the questionnaire, and when couched in general terms 47% of respondents agreed with the idea of such a law, compared with 10% when the question was formulated in the more concrete terms of a clinical situation with alternatives. This result is particularly interesting since it bears witness to the fragility of an opinion expressed in general terms and to the need for ethical reflection before responding. Various factors can influence the wish to speed the death of a patient in order to shorten his or her suffering. One that is certainly crucial is the dividing line between the administration of lethal drugs and the use of terminal sedation such as that proposed in the conclusions to Professor Sicard’s report [16]. The philosophical doctrine of double effect is often adduced to distinguish situations where the administration of sedatives or analgesics or both primarily to increase patient comfort can, in certain situations, hasten the process of death [17]. This is clearly distinct from the voluntary injection of lethal drugs. There is a broad consensus for recommendation of the administration of sedatives and analgesics at the end of life whenever it seems necessary, since the priority is patient comfort [18]. In this situation, the formal intention is to alleviate the patient’s suffering and to increase his or her apparent comfort, death not being the primary aim. Terminal sedation has been widely discussed in the literature [17,19]. Some authors consider that its intention is to accelerate the process of death [17]. This attitude is justified by the lack of absolute certainty regarding patient comfort [20] and by the ordeal experienced by loved ones, and even caregivers, when death is slow in coming. In such situations, the distinction between the wish to ensure the patient’s comfort and the act of euthanasia is fuzzy. The ethics committee of the SRLF recently offered ethical thoughts on persistent signs of agony at the end of life in the ICU [21]. The purity of the intention can seem unrealistic and it is impossible to prove a ‘good intention’. Lastly, the temptation to implement euthanasia may be encouraged by the fact that a majority of public opinion is in favor of euthanasia [22]. Here too, the interpretation of an opinion unsubstantiated by real understanding of the problem is problematic, particularly as the potential risks of euthanasia legislation regarding vulnerable patients are rarely spelled out. In a rare factual study, analysis of the medical records of 761 patients who died in French hospitals showed that 0.9% of conscious patients explicitly requested euthanasia [23]. Recently, during the interviews that were conducted under Professor Sicard’s report, the French Ethics Committee was asked to give its point of view about a possible specific law on euthanasia in France [24]. A large majority, but not all of the committee members, argued against such a law. Whatever their thinking, their analysis clearly specified that euthanasia or assisted suicide should only concern patients without altered consciousness in the terminal phase of a severe disease. Thus, ICU patients would seldom be concerned by such a law. The French Ethics Committee also agreed that, in certain conditions, terminal sedation goes beyond the double effect, given that prescription to alleviate suffering may also hasten death [24].

### Doctor-paramedic differences

In the present study, the answers of the paramedics were overall quite close to those of the doctors. Certain differences should, however, be pointed out. In particular, among the difficulties reported in the application of Leonetti’s law, over 30% of paramedics considered that collective decision-making is hard to achieve (versus 13% of doctors), and close to 20% of paramedics considered that there is a lack of team discussion, whereas only 3% of doctors did. Such differences between doctors and paramedics have already been highlighted [25].

Clearly, our study has the limitations of a questionnaire survey evaluating intentions rather than facts. The certainty of acquaintance with Leonetti’s Law assessed by the answerers was speculated and was not checked by specific tests. Furthermore, questionnaires can generate intuitive answers and the depth of underlying thought cannot be assessed. So, we cannot conclude from this study that half of doctors and paramedics working in intensive care are in favor of euthanasia. Even though care was taken to formulate neutral questions, the way they were formulated may have had an influence. Additionally, it was not possible to speculate on the potential differences between responders and non-responders, knowing that highly committed individuals may have answered preferentially. Finally, only one question referred to a real clinical situation. The answers to this question give a more nuanced picture of the desire for a law authorizing administration of lethal drugs in certain cases.

## Conclusion

This questionnaire survey among intensive care personnel-doctors and paramedics -suggests that Leonetti’s law on the end of life is widely known and applied in ICUs, even though improvements are needed in its implementation, particularly regarding the involvement of paramedics in discussions preceding the medical decision to withhold or withdraw life-sustaining treatment. Likewise, a large majority of respondents considered that Leonetti’s law, as it stands, allows a response to most end of life situations encountered in intensive care. Despite this, one quarter of the respondents would be in favor of the authorization to administer lethal drugs in rare intensive care situations when palliative care is well-managed. Sixty percent of doctors and 50% of paramedics reported having received such a request from patients’ loved ones.

## Abbreviations

ICU: intensive care unit; SRLF: Société de Réanimation de Langue Française (French society of critical care).

## Competing interests

The authors declare that they have no competing interests.

## Author’s contributions

RR, LS, LH, JP G, BE, DD conceived the study, and participated in its design. RR coordinated the study, was responsible for statistical analysis and drafted the manuscript. DD critically revised the manuscript for important intellectual content. All authors read and approved the final manuscript.

## Supplementary Material

Additional file 1 Questionnaire. Click here for file
