# Supplementary material for: End of life in the intensive care unit: should French law be adapted?
Source: Ann Intensive Care. 2014 Feb 25;4:6. doi: 10.1186/2110-5820-4-6 (PMC4015966; doi:10.1186/2110-5820-4-6)
Supplement: Additional file 1 — Questionnaire. [file 2110-5820-4-6-S1.doc]

Additional file: Questionnaire

Question 1: Are you familiar with Leonetti’s law on endoflife issues?

Very □ Quite □ Not very □ Not at all □

Question 2: In the department where you work, do you think that the great majority of physicians are familiar with Leonetti’s law on endoflife issues?

Certainly □ Probably □ Probably not □ No □

Question 3: In the department where you work, do you think that the great majority of registered nurses are familiar with Leonetti’s law on endoflife issues?

Certainly □ Probably □ Probably not □ No □

Question 4: In the department where you work, do you think that Leonetti’s law on the end of life is applied when making decisions to withhold or withdraw life-sustaining treatment?

Always and fully □ Often or incompletely □ Sometimes □ Rarely□ Never □

Question 5: If not, why?

No discussion with the medical and nursing team □

Collective decision-making is hard to achieve □

No discussion with the family □

No external consultant (not called or called but no one available) □

Question 6: In the unit where you work, do you think that Leonetti’s law enables satisfactory decisions to be taken in the great majority of cases of withholding or withdrawing life-sustaining treatment?

Yes □ No □

Question 7: In 2012, during your work in intensive care, were you confronted by a situation where a patient insistently or repeatedly asked you to help him/her die? (‘deliberately hastening death’ not ‘allowing to die’)

No □ Yes, once □ Yes, more than once □

Question 8: In 2012, during your work in intensive care, were you confronted by a situation where a patient’s family insistently or repeatedly asked you to help him/her die or to accelerate the dying process? (‘deliberately hastening death’ not ‘allowing to die’)

No □ Yes, once □ Yes, more than once □

Question 9: In 2012, during your work in intensive care, were you confronted by a situation where a patient’s family clearly and persistently opposed the possibility of treatment withdrawal while such withdrawal seemed reasonable to the whole care team?

No □ Yes, once □ Yes, more than once □

Question 10: In 2012, were you confronted by situations where you would have wished to accelerate the dying process in a patient by injecting lethal drugs? (‘deliberately hastening death’ not ‘allowing to die’)

Yes □ No □

Question 11: In general, in intensive care units, do you think that application of Leonetti’s law enables a response to all endoflife situations?

Always □ Usually □ Not often enough □ Rarely □ Don’t know □

Question 12: In general, in palliative care units, do you think that application of Leonetti’s law enables a response to all endoflife situations?

Always □ Usually □ Not often enough □ Rarely □ Don’t know □

Question 13: In general, in a hospital setting (apart from intensive care and palliative care), do you think that application of Leonetti’s law enables a response to all endoflife situations?

Always □ Usually □ Not often enough □ Rarely □ Don’t know □

Question 14; If you think (previous questions) that Leonetti’s law does not solve all problems, why is that (more than one answer possible)?

It is insufficiently known □ It does not address certain problems □ Decision has to be taken quickly □

Other (specify)………………………………………………………………………………….

Question 15: In an intensive care setting, including well-managed palliative care, would you be in favor of a law authorizing requests for euthanasia**?*

Yes □

Probably □

Yes, but I think it’s impossible to define the legal framework for its application □

No, because it’s impossible to define the legal framework for its application □

Yes, but I think it would represent a slippery slope with a high potential for abuse □

No, because I think the potential for abuse is too great □

Probably not □

No □

*Request for euthanasia:extract from national ethical committee statement 63;

[Translated] ‘Deliberately killing, whatever the circumstances and justifications, remains a transgression. Voluntary euthanasia should continue to be subject to legal authority. But the case should be accorded a special review if its author presents it as voluntary euthanasia. A sort ofrequest for euthanasia,which could be provided for in law, would enable a review of both the exceptional circumstances that could lead to voluntary euthanasia and the conditions for performing it. When the case begins to be investigated or debated, it should be the subject of examination by an interdisciplinary commission whose task would be to evaluate the probity of the claims made by those concerned, not so much regarding their guilt in fact and in law, but as regards their motivation, that is concern to end suffering, respect for a request made by the patient, compassion in the face of the inevitable. The judge, of course, takes the final decision.

Question 16: For palliative care units, including when this care is well managed, would you be in favor of a law authorizing requests for euthanasia?

Yes □

Probably □

Yes, but I think it’s impossible to define the legal framework for its application □

No, because it’s impossible to define the legal framework for its application □

Yes, but I think it would represent a slippery slope with a high potential for abuse □

No, because I think the potential for abuse is too great □

Probably not □

No □

Question 17: For short-stay or follow-up care units (apart from intensive care units or palliative care units), when palliative care is well managed, would you be in favor of a law authorizing requests for euthanasia?

Yes □

Probably □

Yes, but I think it’s impossible to define the legal framework for its application □

No, because it’s impossible to define the legal framework for its application □

Yes, but I think it would represent a slippery slope with a high potential for abuse □

No, because I think the potential for abuse is too great □

Probably not □

No □

Question 18: Overall, would you be in favor of a law authorizing in very precise conditions the administration of lethal drugs on request from a patient with an incurable disease and in whom palliative care would not be effective or would be refused?

Yes □ No □

Question 19: Overall, would you be in favor of a law authorizing in very precise conditions the administration of lethal drugs to an unconscious patient whose dying process is prolonged?

Yes □ No □

Question 20: What is your view regarding the case of a patient whose life-sustaining treatment has been withdrawn (no mechanical ventilation, no artificial kidney, no vasopressor therapy…) and whose death is impending, if death does not occur after a certain time (one answer only)?

It’s not a problem provided the patient is comfortable. Death comes when it must, however long it takes □

You hope death comes quickly, but you do nothing else □

You hope death comes quickly and you increase the doses of sedatives even if the patient is comfortable □

You hope death comes quickly and you administer a lethal drug (curare) despite legal prohibition □

You hope death comes quickly, and you would be in favor of the administration of a lethal drug provided there is a legal framework for this practice □

Question 21: In which situations do you think that the administration of lethal drugs can be considered (more than one answer possible)?

None □

At the request of a patient who is suffering from pain uncontrolled by optimal symptomatic

treatment and whose disease is incurable □

When, despite well-managed palliative care, a non-institutionalized patient continues

to ask for a quick death □

When, despite well-managed palliative care, an institutionalized patient continues

to ask for a quick death □

When a patient with locked-in syndrome who has not recovered after several months asks you to endhis/her life □

When it has been decided to withdraw treatment from an unconscious intensive care patient whosecomfort care is optimized and whose death does not occur after a certain time (for example, 48 hours) □

I do not wish to answer this question □

Question 22: Do you think that it is technically possible to draw up a law on voluntary euthanasia that authorizes the administration of lethal drugs and which protects against potential abuse?

Applicable to a conscious patient

Yes □ No □ Don’t know □

Applicable to an unconscious patient

Yes □ No □ Don’t know □
